# The association between prenatal exposure to mixed air pollutants and birth defects risk: a population-based study in Tangshan, China

**DOI:** 10.3389/fpubh.2026.1791132

**Published:** 2026-04-13

**Authors:** Min Guo, Yuelin Li, Haishuang Li, Yuxia Yang, Hongzhen Zhang

**Affiliations:** 1 Department of Obstetrics, Tangshan Maternal and Children Health Hospital, Tangshan, Hebei, China; 2 School of Public Health, North China University of Science and Technology, Tangshan, Hebei, China; 3 Department of Obstetrics and Gynecology, The First Hospital of Hebei Medical University, Shijiazhuang, Hebei, China

**Keywords:** air pollutants, Bayesian kernel machine regression, birth defects, joint effect, prenatal exposure, quantile g–computation

## Abstract

**Background:**

Birth defects (BDs) are a major health concern worldwide, with air pollution being a key environmental risk factor. Current evidence, especially from industrial regions and regarding multi-pollutant exposure, is limited. This study examined the association between prenatal exposure to mixed air pollutants from preconception to early pregnancy and the risk of BDs in Tangshan, China.

**Methods:**

We conducted a population-based study using data from a regional maternal and child health surveillance system. Maternal exposure to air pollutants (PM_10_, PM_2.5_, SO_2_, NO_2_, CO, and O_3_) from 12 weeks before pregnancy to gestational week 13 was assessed using inverse distance weighting interpolation. Multi-pollutant statistical models, including quantile g-computation (QGC) and Bayesian kernel machine regression (BKMR), were employed to analyze the association with offspring BDs while adjusting for covariates.

**Results:**

This analysis included 19,053 mother–offspring pairs, with 540 cases of BDs identified (28.34 per 1,000, 95%CI: 25.99–30.70). Using QGC, a 19% higher risk of BDs was observed per quartile increase in multi-pollutant mixture (OR = 1.19, 95%CI: 1.01–1.41, *P* = 0.039). The positive weights for individual pollutants were as follows: PM_2.5_ (0.287), SO_2_ (0.235), O_3_ (0.194), NO_2_ (0.149), and PM_10_ (0.135). BKMR revealed a nonlinear positive joint effect, with the strength of the association increasing at higher exposure levels. All pollutants except CO contributed positively to the joint effect, and potential interactions were observed between PM_10_-SO_2_, NO_2_-SO_2_, NO_2_-O_3_, and CO-O_3_. No significant effect modification by residential area or offspring sex was found in QGC; however, in subgroup analysis, O_3_ was the key pollutant in rural areas and SO_2_ in female offspring. Furthermore, BKMR showed a significant positive association in all subgroups when the mixture exposure level exceeded the median.

**Conclusion:**

The findings suggest that periconceptional exposure to mixed air pollutants is associated with an increased risk of BDs, driven primarily by PM_2.5_, SO_2_, and O_3_. Thus, these results provide a foundation for developing targeted air pollution protection strategies for pregnant women, advancing primary prevention of BDs, and guiding the optimization of healthcare resource allocation.

## 1 Introduction

Birth defects (BDs), commonly referred to as congenital anomalies, represent a leading cause of infant mortality and childhood disability worldwide, posing a significant public health challenge (1). It is estimated that approximately 240,000 neonates die from BDs within the first 28 days of life annually, with an additional 170,000 deaths occurring among children aged between 28 days and 5 years (2). Many children born with complex BDs require multiple surgical interventions and long-term comprehensive care from infancy through adulthood. This imposes not only a substantial economic burden on families but also increases the risk of persistent psychological issues and social adaptation difficulties for affected children (3). Consequently, BDs exert a profound and multidimensional negative impact on individual quality of life, family wellbeing, and public health resources.

The etiology of BDs is complex, involving genetic, infectious, nutritional, and environmental factors (4, 5). Previous epidemiological studies have demonstrated that maternal exposure to air pollutants increases the risk of BDs in offspring, including specific subtypes such as congenital heart disease (CHD), polydactyly/syndactyly, and cleft lip/palate (6–9). However, several uncertainties persist in the existing literature. First, due to the high collinearity commonly observed among air pollutants, most studies have relied on single-pollutant models, which fail to systematically assess the health effects of mixed exposure in real-world settings, particularly in countries or regions with elevated air pollution levels (7, 10). Second, from a developmental biology perspective, the 3 months prior to conception represent a critical window for ovarian follicle maturation, while early pregnancy is a crucial period for fetal organ differentiation and development (11, 12). Thus, the exposure window spanning from preconception to early gestation likely represents a sensitive period for air pollution-induced developmental toxicity (13–16). Nevertheless, studies specifically evaluating the association between preconception air pollutant exposure and BDs during this critical window remain scarce (6, 17).

Tangshan, a major industrial city in northern China, has an economy traditionally centered on steel and coal production, historically resulting in severe air pollution. Although pollutant concentrations in the region have decreased in recent years due to economic restructuring and stricter environmental policies, they remain relatively high. Leveraging birth data from this heavily industrialized city, this study employed multi-pollutant statistical models to systematically evaluate the association between prenatal exposure to a mixture of six criteria air pollutants (PM_10_, PM_2.5_, SO_2_, NO_2_, CO, and O_3_) during the period from preconception to early pregnancy and the risk of BDs in offspring (18, 19). The findings are intended to inform strategies for BDs prevention and to guide targeted public health policies in this industrial setting.

## 2 Methods

### 2.1 Study population

**Figure 1:**
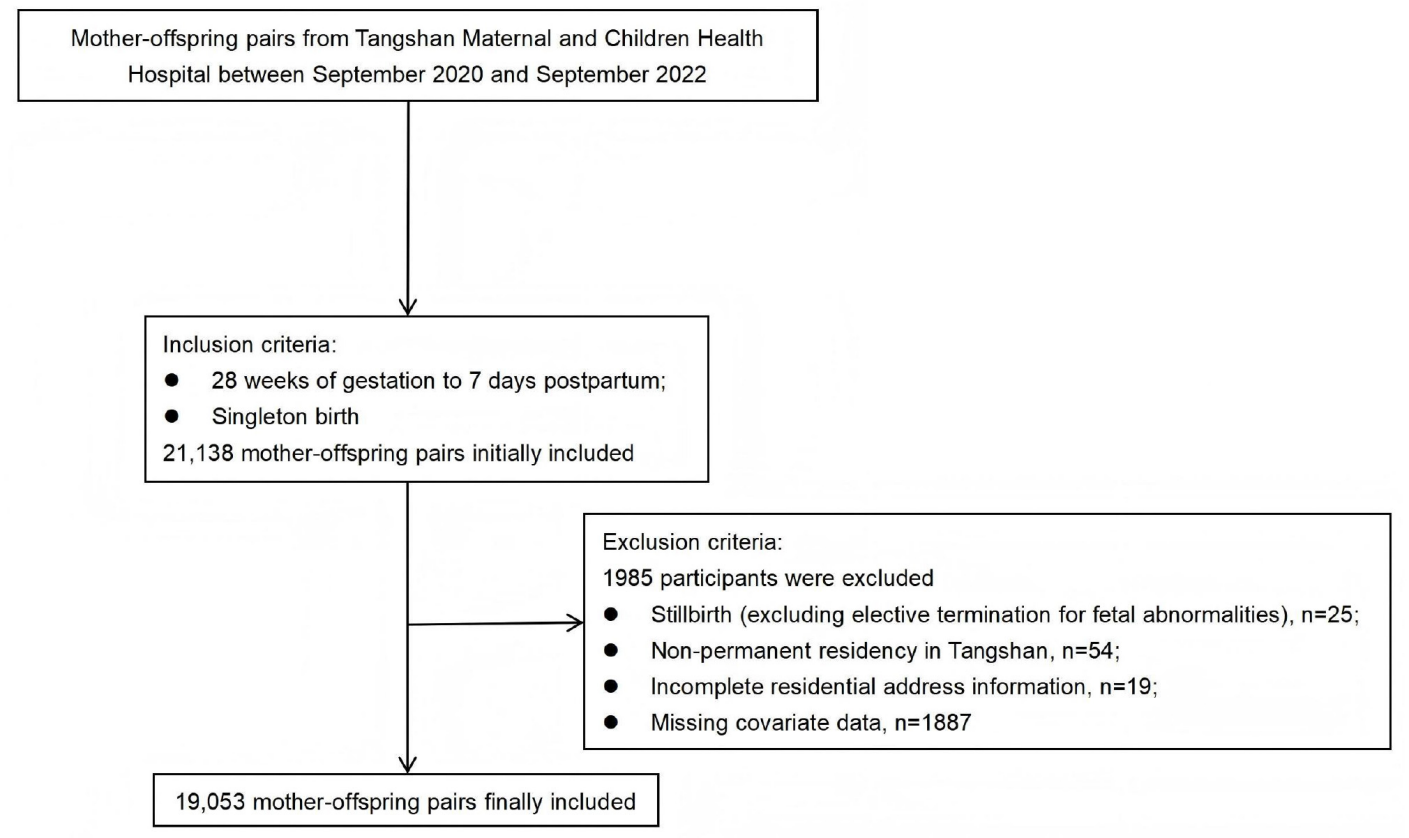
Flowchart of mother-offspring pairs selection.

This population-based retrospective cohort study used data from the Hebei Maternal and Child Health Monitoring and Management System (https://lf.hbfyjk.com/#/login?redirect=/). The criteria for participant inclusion were defined as follows: (1) delivery at a gestational age of 28 weeks or more, with follow-up through 7 days postpartum; (2) singleton birth. Based on these criteria, an initial dataset comprising 21,138 mother-offspring pairs, who delivered at Tangshan Maternal and Children Health Hospital between September 2020 and September 2022, was extracted. Participants were subsequently excluded if they met any of the following conditions: (1) stillbirth (excluding selective termination for fetal abnormalities); (2) non-permanent residency in Tangshan; (3) incomplete residential address information; (4) missing data for key covariates. Following these exclusion criteria, 19,053 mother-offspring pairs were included in the final analysis. The detailed process of participant inclusion and exclusion is illustrated in Figure 1.

Data were obtained on key variables, including maternal residential address (street level), age, educational level, employment status, parity, last menstrual period (LMP), gestational age at delivery, offspring sex, birth weight, and BDs diagnosis.

The study protocol was approved by the Medical Ethics Committee of Tangshan Maternal and Children Health Hospital (No.: 2023-019-01). The requirement for informed consent was waived due to the retrospective analysis of fully anonymized medical records.

### 2.2 Environmental exposure assessment

Air quality data were obtained from the Tangshan Grid-Based Precision Monitoring Platform (https://wgh.aepic.net:9817/), comprising daily concentrations of PM_10_, PM_2.5_, SO_2_, NO_2_, CO, and O_3_ (the latter as the daily maximum 8-hour average) from 30 state- and province-controlled stations across the city between September 2019 and June 2022. Concentrations were reported in μg/m^3^ for all pollutants except CO, which was expressed in mg/m^3^. A summary is provided in Supplementary Table S1.

Geographical coordinates (longitude and latitude) in the WGS 84 coordinate system were derived for the 30 monitoring stations as well as for the street-level residential addresses of the 19,053 participants, using the Google Geocoding API (https://code.google.com/apis/console/). The spatial distributions of the monitoring stations and the participants were subsequently visualized through mapping in ArcGIS 10.8 software (ESRI, Redlands, CA, USA), as presented in Supplementary Figures S1, S2.

Individual exposure was estimated using the inverse distance weighting (IDW), a widely applied spatial interpolation technique in environmental health studies (20–22). Briefly, the Euclidean distance between each participant's residential address and nearby air quality monitoring stations was calculated based on geocoded coordinates. Daily individual exposure levels for the six pollutants were then estimated by IDW, with weights equal to the inverse squared distance (1/d^2^), using monitoring stations located within a 50 km radius of the residence (23–25).

The exposure window was defined as the period from 12 weeks before pregnancy to gestational week 13, using the date of the LMP as the reference point (Supplementary Figure S3). For each pollutant, the average exposure level during this window was calculated for each participant and used as the exposure variable in subsequent analyses.

### 2.3 Outcome definition

BDs were defined as structural, functional, or metabolic abnormalities originating during embryogenesis (1). In accordance with the Maternal and Child Health Surveillance Manual of China and the Chinese version of the International Classification of Diseases, 11th Revision (ICD-11), BDs were classified using codes Q00 to Q99. Detailed information on specific categories and corresponding codes is provided in Supplementary Table S2.

As a provincial-level sentinel hospital for BDs surveillance, Tangshan Maternal and Children Health Hospital routinely monitors cases among perinatal infants from 28 weeks of gestation to 7 days postpartum. Diagnoses were established through single or combined methods, including physical examination, ultrasound, chromosomal and genetic testing, and biochemical tests when indicated. All confirmed cases were recorded on standard Sentinel Site Birth Defect Case Report and Hospital Delivery Report Forms by trained staff. These records underwent administrative review and quality control before quarterly submission to the Hebei Provincial Maternal and Child Health Center.

The unit of analysis was the individual neonate. For neonates with multiple BDs, each infant was counted as a single case in the primary analysis of total BDs (i.e., the unit of count was “per neonate”), to assess the risk of neonates presenting with at least one defect. In sensitivity analyses, isolated BDs (defined as neonates presenting with only one type of BD) were further examined as a secondary outcome measure.

### 2.4 Covariates

Based on prior literature and clinical relevance, the following covariates were selected as potential confounders: maternal age (< 25, 25–29, 30–34, or ≥35 years), educational level ( ≤ 9, 10–12, or ≥13 years), employment status (employed/unemployed), residential area (urban/rural), parity (0, 1, or ≥2), season of conception (spring: March-May, summer: June-August, autumn: September-November, winter: December-February), gestational age (< 37 or ≥37 weeks), and offspring sex (male/female). All listed covariates were included in multivariable-adjusted models in the subsequent analyses.

### 2.5 Statistical analysis

In descriptive analyses, categorical variables were presented as frequencies (percentages), with between-group comparisons performed using the chi–square (χ^2^) test. Exposure levels to the six air pollutants during the exposure window were summarized using percentiles, and correlations among the pollutants were assessed via Spearman rank correlation analysis.

In the primary analysis, the association between exposure to single-pollutant and the risk of BDs was first examined. Both unadjusted and adjusted logistic regression models were fitted. The effect estimate was expressed as odds ratio (OR) and 95% confidence intervals (CI) per 10 μg/m^3^ increase in PM_10_, PM_2.5_, SO_2_, NO_2_, and O_3_, and per 0.1 mg/m^3^ increase in CO. To visually evaluate potential nonlinear exposure-response (E-R) relationships, restricted cubic spline (RCS) models were constructed for each pollutant across its full concentration range, with knots placed at *P*_10_, *P*_50_ (reference), and *P*_90_. All models were adjusted for potential confounders.

To systematically evaluate the health effects of exposure to mixed air pollutants, quantile g-computation (QGC) and Bayesian kernel machine regression (BKMR) were employed as complementary approaches within different statistical frameworks. QGC is a mixture analysis method based on parametric generalized linear models. This approach does not prespecify the direction of the effect for each pollutant and can simultaneously estimate the joint effect and identify key toxic components. Its main advantages include intuitive interpretation, ease of communication, and high computational efficiency (26). In this analysis, all six pollutants were included as continuous variables at their original concentrations. The result was expressed as OR and 95% CI corresponding to an increase of one quartile range in the mixture, along with the positive or negative contribution weight of each pollutant.

BKMR is a nonparametric Bayesian statistical framework that flexibly fits E-R surfaces using Gaussian kernel functions. This approach captures nonlinear relationships and potential interactions among pollutants without imposing linearity or additivity assumptions. It comprehensively accounts for parameter uncertainty via Markov chain Monte Carlo (MCMC) algorithms, enhancing result reliability (27). Prior to model fitting, all pollutant concentrations were log-transformed and normalized to reduce skewness and ensure comparability of scales. Given the large sample size, the model was run with 1,000 iterations. BKMR outputs included: (1) Joint effect: The estimated risk of BDs was computed as the multi-pollutant exposure increased from *P*_10_ to *P*_90_ in 5% increments, with constituent pollutants fixed at their *P*_50_ as the reference. (2) Single–pollutant effect: The change in risk was estimated when a target pollutant increased from *P*_25_ to *P*_75_, while holding all other pollutants at their *P*_25_, *P*_50_, or *P*_75_. (3) Univariate E-R relationship: The E-R curve for a specific pollutant was plotted with all other pollutants fixed at their median. (4) Bivariate E-R relationship: The E-R curve of one pollutant was examined for shifts when a second pollutant was fixed at low (*P*_25_), median (*P*_50_), or high (*P*_75_) levels, with all others at their median; curve crossings suggest potential interaction.

To assess the robustness of the main findings, subgroup and sensitivity analyses were performed. Effect modification by residential area and offspring sex was assessed by incorporating interaction terms into the QGC. For BKMR, which does not readily accommodate such product terms, only stratified analyses were examined to evaluate the joint effect within subgroups. A series of sensitivity analyses were then conducted: (1) restriction to isolated BDs cases (n=19,034); (2) application of weighted quantile sum (WQS) regression, in which pollutant effects were constrained to be positive based on earlier findings (28); and (3) construction of an environmental risk score using the adaptive network regularization (ANR) method (29, 30). This score was included as a continuous variable in a logistic regression model to estimate the OR and 95%CI associate with per standard deviation increase.

## 3 Results

### 3.1 Characteristics of the study population

The final analysis included 19,053 mother-offspring pairs. Among them, 540 infants were diagnosed with BDs, corresponding to an incidence of 28.34 per 1,000 (95%CI: 25.99–30.70). Demographic characteristics are detailed in Table 1. Offspring with BDs had significantly higher percentages of preterm birth, low birth weight, and macrosomia (all *P* < 0.001), but no significant difference in sex distribution was observed. Their mothers were more likely to be ≥35 years old, have an education level of junior high school or below, be unemployed, reside in rural areas, be multiparous, and have conceived in summer or autumn (all *P* < 0.05).

**Table 1:** Basic characteristics of participants in Tangshan, China.

Characteristics	Total (n, %)	Non-BDs (n, %)	BDs (n, %)	*P*-value
	*N* = 19,053	*n* = 18,513	*n* = 540	
Maternal age, years				
< 25	1,919 (10.07)	1,863 (10.06)	56 (10.37)	0.040
25–29	7,932 (41.63)	7,731 (41.76)	201 (37.22)	
30–34	6,780 (35.58)	6,585 (35.57)	195 (36.11)	
≥35	2,422 (12.71)	2,334 (12.61)	88 (16.30)	
Educational level, years				
≤ 9	3,845 (20.18)	3,714 (20.06)	131 (24.26)	0.039
10–12	2,851 (14.96)	2,781 (15.02)	70 (12.96)	
≥13	12,357 (64.86)	12,018 (64.92)	339 (62.78)	
Employment status				
Employed	18,081 (94.90)	17,581 (94.97)	500 (92.59)	0.013
Unemployed	972 (5.10)	932 (5.03)	40 (7.41)	
Residential area				
Urban	13,699 (71.90)	13,338 (72.05)	361 (66.85)	0.008
Rural	5,354 (28.10)	5,175 (27.95)	179 (33.15)	
Parity				
0	11,272 (59.16)	10,979 (59.30)	293 (54.26)	0.021
1	6,781 (35.59)	6,573 (35.50)	208 (38.52)	
≥2	1,000 (5.25)	961 (5.19)	39 (7.22)	
Season of conception				
Spring	4,843 (25.42)	4,733 (25.57)	110 (20.37)	< 0.001
Summer	4,129 (21.67)	3,973 (21.46)	156 (28.89)	
Autumn	4,882 (25.62)	4,720 (25.50)	162 (30.00)	
Winter	5,199 (27.29)	5,087 (27.48)	112 (20.74)	
Gestational age, weeks				
< 37	2,069 (10.86)	1,970 (10.64)	99 (18.33)	< 0.001
≥37	16,984 (89.14)	16,543 (89.36)	441 (81.67)	
Offspring sex				
Male	9,827 (51.58)	9,544 (51.55)	283 (52.41)	0.695
Female	9,226 (48.42)	8,969 (48.45)	257 (47.59)	
Birth Weight, g				
< 2,500	1,391 (7.30)	1,312 (7.09)	79 (14.63)	< 0.001
2,500–3,999	16,467 (86.43)	16,045 (86.67)	422 (78.15)	
≥4,000	1,195 (6.27)	1,156 (6.24)	39 (7.22)	

BDs, birth defects.

The χ^2^ test was used to compare Non-BDs group and BDs group.

### 3.2 Characteristics of air pollutant exposure

Table 2 displays the distribution of maternal air pollutant exposure during the exposure window. Median (*P*_25_, *P*_75_) concentrations were 87.66 (76.73, 99.60) μg/m^3^ for PM_10_, 43.17 (38.24, 49.03) μg/m^3^ for PM_2.5_, 14.46 (10.17, 16.85) μg/m^3^ for SO_2_, 42.16 (37.20, 46.20) μg/m^3^ for NO_2_, 1.06 (0.93, 1.18) mg/m^3^ for CO, and 90.66 (71.54, 113.07) μg/m^3^ for O_3_. Notably, median exposure to PM_10_, PM_2.5_, and NO_2_ exceeded the World Health Organization Air Quality Guidelines 2021 (AQG2021:15 μg/m^3^ for PM_10_, 5 μg/m^3^ for PM_2.5_, and 10 μg/m^3^ for NO_2_) by 5.84–, 8.63–, and 4.22–fold, respectively (31). As shown in Supplementary Figure S4, moderate to strong significant positive correlations were observed among PM_10_, PM_2.5_, SO_2_, NO_2_, and CO, with correlation coefficients ranging from 0.58 to 0.82 (all *P* < 0.001). Conversely, O_3_ was negatively correlated with the other pollutants (*r* = −0.48 to −0.07, all *P* < 0.001).

**Table 2:** Distribution of air pollutants during the exposure assessment period.

Air pollutant	Min	*P_10_*	*P_25_*	*P_50_*	*P_75_*	*P_90_*	Max
PM_10_, μg/m^3^	47.73	68.51	76.73	87.66	99.60	105.83	137.80
PM_2.5_, μg/m^3^	22.03	34.49	38.24	43.17	49.03	54.04	73.84
SO_2_, μg/m^3^	4.36	8.05	10.17	14.46	16.85	19.36	35.29
NO_2_, μg/m^3^	19.06	34.11	37.20	42.16	46.20	49.26	58.94
CO, mg/m^3^	0.53	0.83	0.93	1.06	1.18	1.40	2.24
O_3_, μg/m^3^	52.06	65.95	71.54	90.66	113.07	124.80	141.87

### 3.3 Association between prenatal exposure to single-pollutant and BDs risk

Supplementary Table S3 summarizes the findings from the logistic regression. After adjustment, each 10 μg/m^3^ increase in PM_10_ (OR = 1.14, 95%CI: 1.06–1.23), PM_2.5_ (OR = 1.25, 95%CI: 1.10–1.45) and NO_2_ (OR = 1.22, 95%CI: 1.04–1.42) was positively associated with BDs risk. Multivariable-adjusted RCS identified significant nonlinear positive E-R relationships for PM_10_, PM_2.5_, SO_2_, and NO_2_ with BDs (*P* for overall < 0.05, *P* for nonlinearity < 0.05), as presented in Supplementary Figure S5.

### 3.4 Association between prenatal exposure to mixed air pollutants and BDs risk

**Figure 2:**
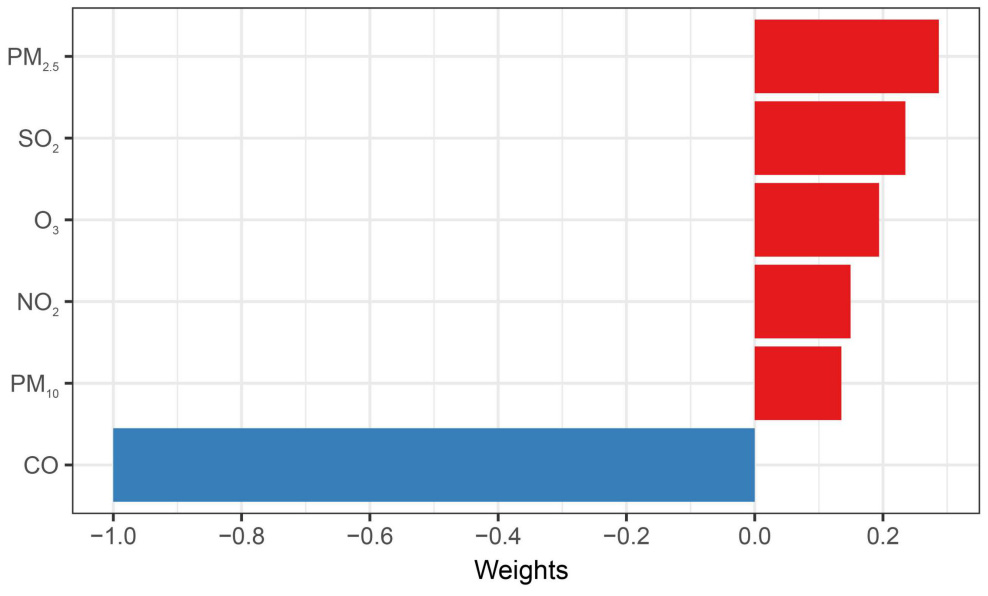
Pollutant-specific weights to the joint effect for BDs risk using quantile g-computation (QGC). Model was adjusted for maternal age, educational level, employment status, residential area, parity, season of conception, gestational age, offspring sex. BDs, birth defects.

Results from the QGC analysis are shown in Figure 2 and Supplementary Table S4. A positive association was observed, with an OR of 1.19 (95%CI: 1.01–1.41, *P* = 0.039) per quartile increase in multi-pollutant mixture. The positive weights of individual pollutants contributing to the joint effect were: PM_2.5_ (0.287), SO_2_ (0.235), O_3_ (0.194), NO_2_ (0.149), and PM_10_ (0.135), whereas CO exhibited a negative weight (1.00).

**Figure 3:**
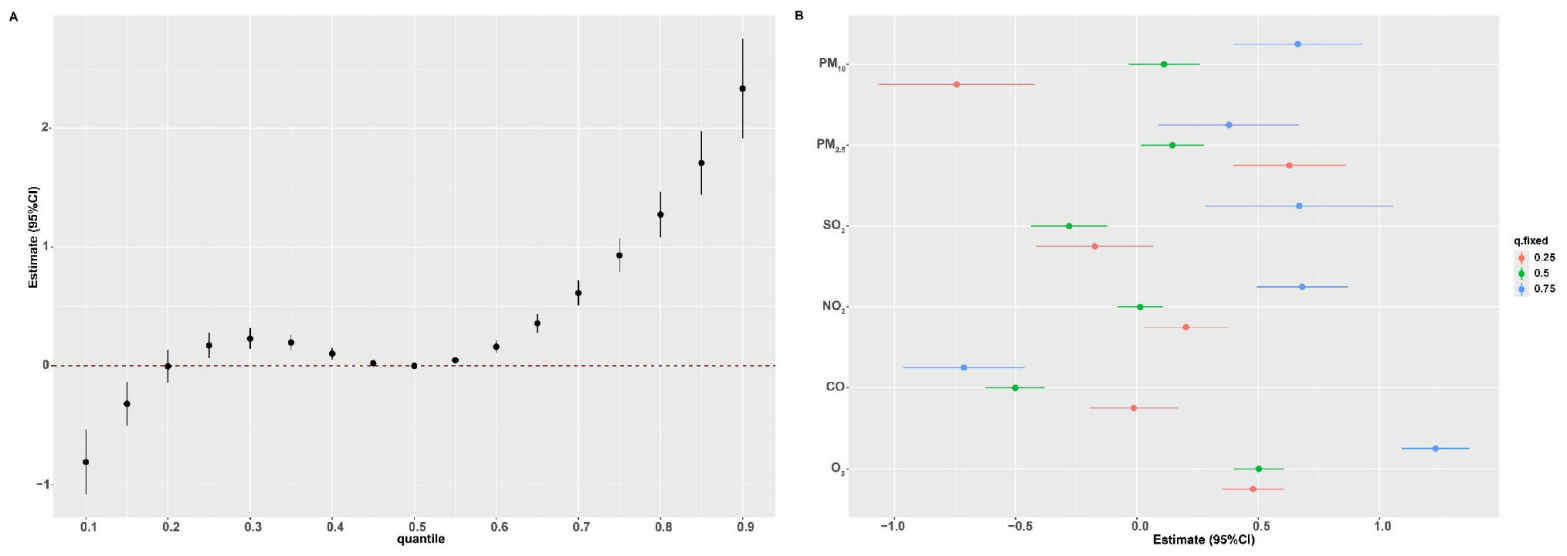
Association of mixed air pollutants exposure with BDs risk estimated using Bayesian kernel machine regression (BKMR). Air pollutants concentrations were ln-transformed and normalized. The estimated change of multi-pollutant mixture exposure on BDs risk when all air pollutants were at a particular quantile compared to *P*_50_ **(A)**. Single-pollutant exposure on BDs risk when the target air pollutant was at *P*_75_ versus *P*_25_, while all other air pollutants were fixed at either *P*_25_, *P*_50_, or *P*_75_ **(B)**. Models were adjusted for maternal age, educational level, employment status, residential area, parity, season of conception, gestational age, offspring sex. BDs, birth defects; CI, confidence interval.

**Figure 4:**
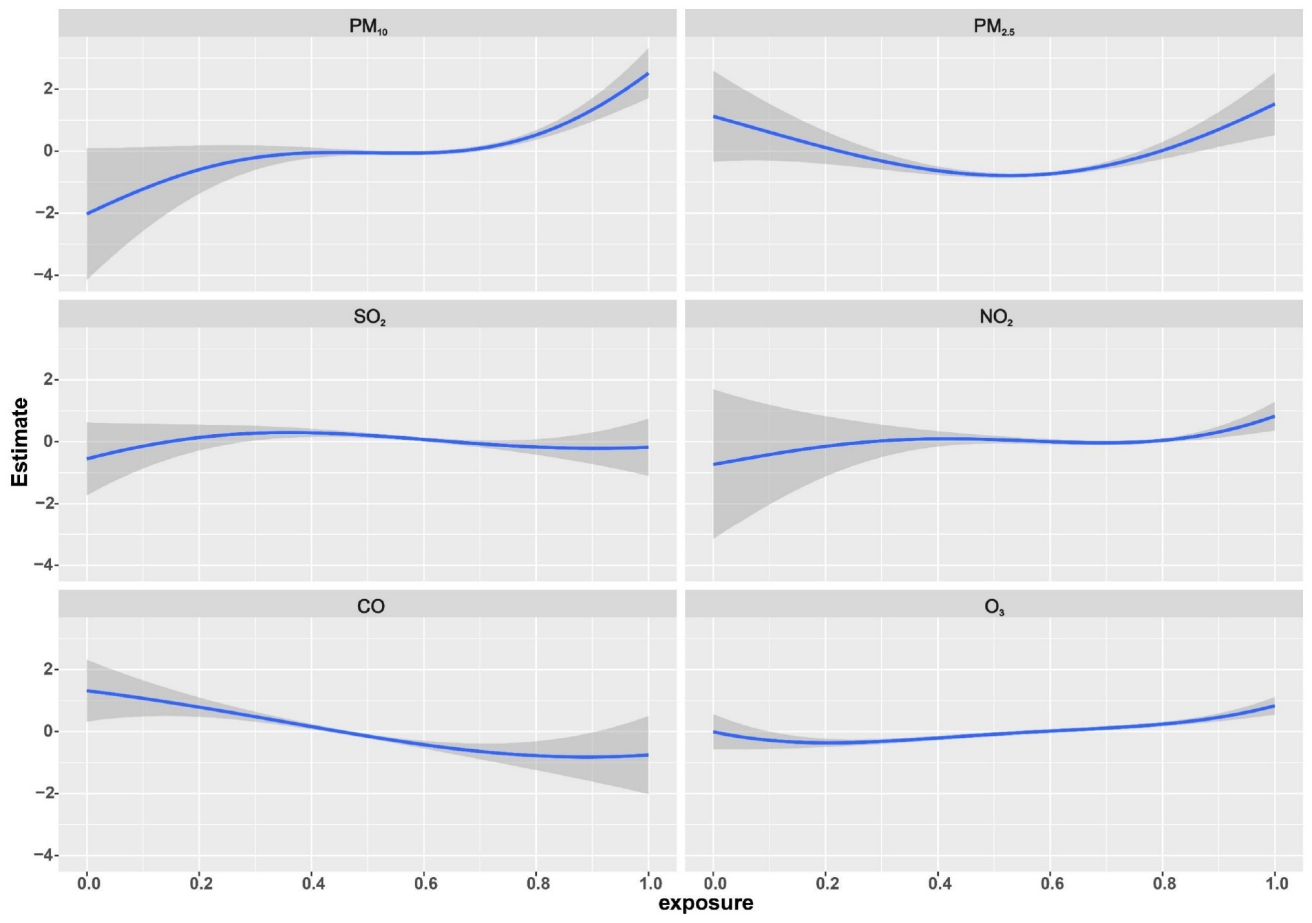
Univariate exposure-response relationship with BDs risk using Bayesian kernel machine regression (BKMR). Curves were plotted for each air pollutant with the other air pollutants fixed at *P*_50_. Gray shading represented 95% credible intervals. Air pollutants concentrations were ln-transformed and normalized. Models were adjusted for maternal age, educational level, employment status, residential area, parity, season of conception, gestational age, offspring sex. BDs, birth defects.

**Figure 5:**
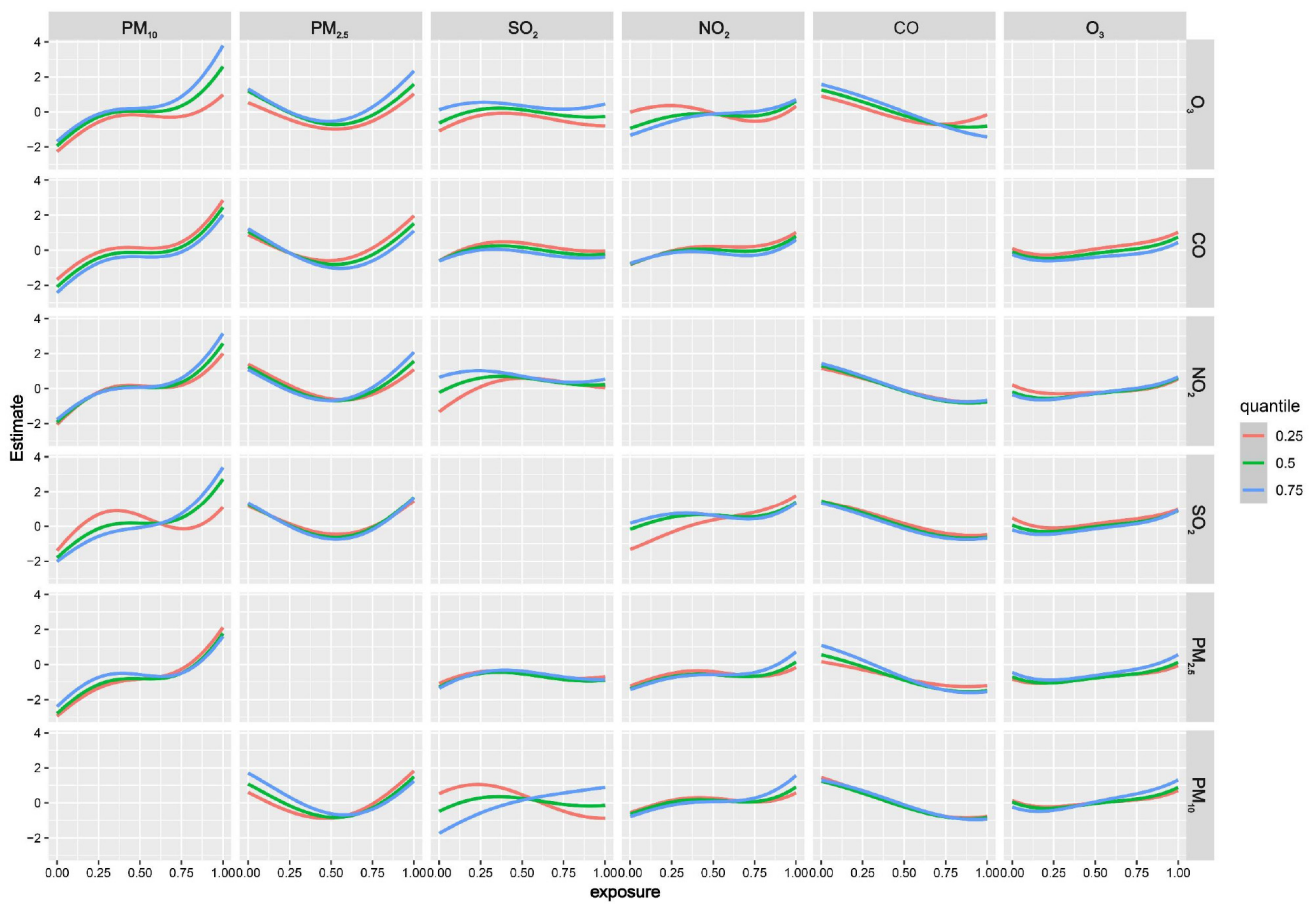
Bivariate exposure-response relationship with BDs risk using Bayesian kernel machine regression (BKMR). Curves were plotted for each pair of air pollutants by fixing another air pollutant at *P*_25_, *P*_50_ and *P*_75_ and all other pollutants at their *P*_50_. Air pollutants concentrations were ln-transformed and normalized. Models were adjusted for maternal age, educational level, employment status, residential area, parity, season of conception, gestational age, offspring sex. BDs, birth defects.

BKMR analysis found a nonlinear positive association between multi-pollutant exposure and BDs risk. When all pollutants reached or exceeded *P*_55_, the risk showed an increasing trend that was statistically significant (Figure 3A). This finding was consistent with the results from the QGC analysis. The model further quantified the independent effect of individual pollutants under varying background exposure. As illustrated in Figure 3B, PM_2.5_ and O_3_ were consistently positively associated with BDs risk, whereas PM_10_ and SO_2_ were positive only at high co-exposure levels. NO_2_ was significantly positively associated when the remaining pollutants were at *P*_25_ or *P*_75_, and CO was negatively associated at median-to-high backgrounds. Within these significant associations, the effect strength tended to increase with higher background exposure levels. Furthermore, with all other pollutants held at their median, univariate E-R curves confirmed positive nonlinear shapes for PM_10_ and PM_2.5_ and a negative linear shape for CO (Figure 4). Finally, analysis of bivariate E-R curves suggested potential interactions between PM_10_-SO_2_, NO_2_-SO_2_, NO_2_-O_3_, and CO-O_3_ (Figure 5).

### 3.5 Subgroup and sensitivity analyses

**Figure 6:**
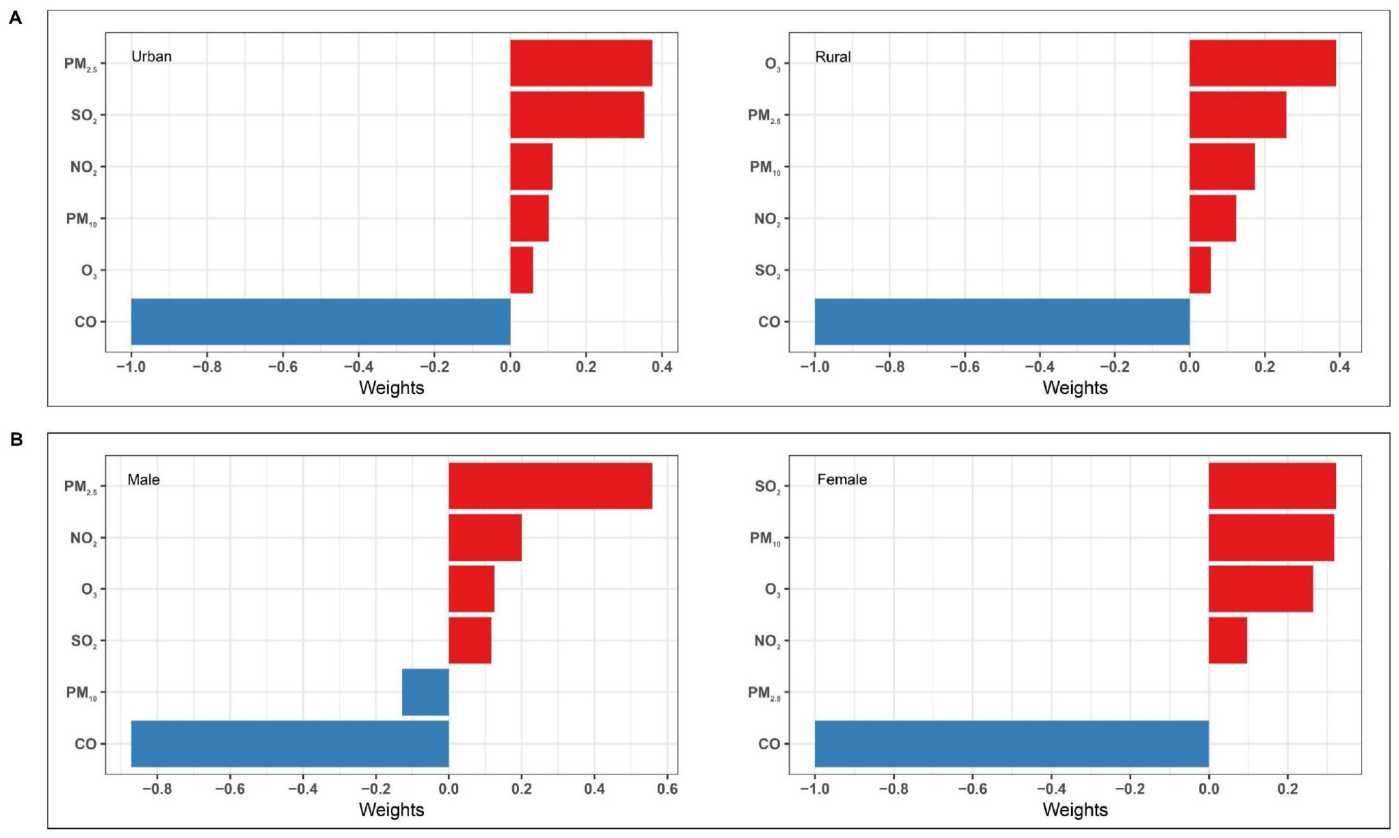
Pollutant-specific weights to joint effect for BDs risk using quantile g-computation (QGC) by subgroup. Analyses were stratified by residential area **(A)** and offspring sex **(B)**. Models **(A)** were adjusted for maternal age, educational level, employment status, parity, season of conception, gestational age, offspring sex. Models **(B)** were adjusted for maternal age, educational level, employment status, residential area, parity, season of conception, gestational age. BDs, birth defects.

**Figure 7:**
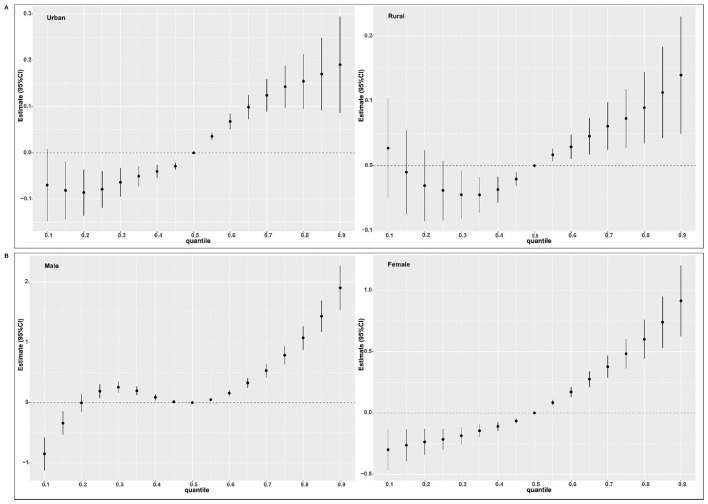
Association of mixed air pollutants exposure with BDs risk using Bayesian kernel machine regression (BKMR) by subgroup. Analyses were stratified by residential area **(A)** and offspring sex **(B)**. Air pollutants concentrations were ln-transformed and normalized. The estimated change of multi-pollutant mixture on BDs risk when all air pollutants were at a particular quantile compared to *P*_50_. Models **(A)** were adjusted for maternal age, educational level, employment status, parity, season of conception, gestational age, offspring sex. Models **(B)** were adjusted for maternal age, educational level, employment status, residential area, parity, season of conception, gestational age. BDs, birth defects; CI, confidence interval.

Subgroup analyses revealed no statistically significant effect modification by residential area or offspring sex on the association in QGC analysis (all *P* for interaction > 0.05; Supplementary Figure S6). Nonetheless, a significant positive joint effect was detected in the rural residence subgroup (with O_3_ as the key driver) and the female offspring subgroup (with SO_2_ as the key driver), shown in Figure 6. Consistent with main analysis, BKMR stratified analyses indicated that in every subgroup, the multi-pollutant mixture was significantly positively associated with BDs risk once the exposure level was at or above *P*_55_ (Figure 7).

The association remained robust in sensitivity analyses confined to isolated BDs (Supplementary Figure S7 and Supplementary Table S4). Additionally, findings from the WQS and ANR-based logistic regression models confirmed the direction and significance of the primary analysis (Supplementary Table S4).

## 4 Discussion

This population-based retrospective cohort study in Tangshan, China, found that prenatal exposure to mixed air pollutant before and during early pregnancy was consistently associated with an increased risk of BDs in offspring, particularly at higher exposure levels. Although PM_2.5_ was the primary driver of this joint effect in the overall population, O_3_ and SO_2_ emerged as the key drivers in the rural residence and female offspring subgroups, respectively. These findings provide new insights into the complex relationship between air pollution and BDs. They underscore the necessity of implementing protective measures for women during the preconception and early pregnancy period in heavily polluted regions and highlight the importance of tailoring public health interventions to specific population subgroups.

In this study, we observed that the highest proportion of BDs was observed among pregnancies conceived in autumn, followed by those conceived in summer. Although a winter pollution peak exists in the Tangshan region for most pollutants (with the exception of O_3_; Supplementary Figure S8), this discrepancy may be explained by two main factors. One involves the definition of the exposure window, which was based on each individual's LMP (from 12 weeks before pregnancy to gestational week 13) rather than fixed calendar periods. Consequently, when the number of BDs is analyzed by conception season, the actual calendar months covered by the exposure window vary across individuals within the same season group. This individual-level alignment may attenuate seasonal peaks that would otherwise appear in time-series analyses using fixed calendar intervals. More importantly, the alignment of critical developmental windows with seasonal pollution patterns provides a biologically plausible explanation for this finding (12). For pregnancies conceived in autumn, the critical period for organogenesis coincides with autumn and winter. This finding suggests that the health effects of air pollution depend not only on average exposure levels but also on the timing of exposure relative to critical developmental windows.

A central methodological challenge in assessing the health effects of multi-pollutant mixture lies in addressing the high collinearity among pollutants to identify potential synergistic, additive, or antagonistic effects. In recent years, novel statistical models such as BKMR and QGC have been developed and applied in epidemiological research, providing essential tools for disentangling joint effects (32). Nevertheless, studies focusing specifically on the association between mixed air pollutant exposure and BDs remain relatively scarce. A study based on the Guangdong CHD Registry, which encompassed 40 medical institutions across 21 cities, included 6,038 CHD patients and 5,227 healthy controls and employed the QGC approach (10). It reported a monotonic increase in the risk of CHD with rising mixture exposure, showing a 47% elevation in risk per quartile increment (OR = 1.47, 95%CI: 1.34–1.61). The effect was stronger in early pregnancy than in mid-to-late pregnancy, with PM_10_ contributing the greatest weight. Another study performed in Chongqing, China, evaluated the joint effect of PM_2.5_ constituents on CHD, also applying QGC (7). The findings indicated that co-exposure to PM_2.5_ constituents in early pregnancy raised the overall risk of CHD by 17% (OR = 1.17, 95%CI: 1.03–1.33), with risks for ventricular septal defect, patent ductus arteriosus, and other subtypes increasing by 22%, 29%, and 33%, respectively. Preconceptional exposure was associated with a 38% higher risk of patent ductus arteriosus (OR = 1.38, 95% CI: 1.07–1.78). The authors further highlighted that the marginal mixture OR for the joint effect of constituents during both the preconceptional and early pregnancy period exceeded that for PM_2.5_ alone, suggesting that PM_2.5_ toxicity may be driven by synergistic actions of its components. In this study, we used multiple analytical strategies to address multicollinearity and systematically examined the association between exposure to mixed air pollutants from 12 weeks before pregnancy through gestational week 13 and the risk of BDs. Results from multiple models consistently showed that such co-exposure was significantly associated with an increased risk of BDs. Specifically, QGC estimated a 19% increase in the risk of BDs per quartile rise in the mixture exposure (OR = 1.19, 95% CI: 1.01–1.41). Moreover, BKMR estimates revealed a significant positive association when exposure to the mixture was above the median level.

To translate the relative risk estimates into publicly relevant quantitative metrics, we calculated the population-attributable risk difference based on the observed baseline incidence of BDs in this study (28.34 per 1,000) and the effect estimate from QGC analysis. The calculation reveals that for each quartile increase in maternal exposure to multi-pollutant mixture before and during early pregnancy is associated with an additional approximately 5.38 cases of BDs per 1,000. This translation of relative risk into an absolute risk difference quantifies the potential additional disease burden, indicating that effectively reducing air pollution in high-exposure industrial cities like Tangshan could prevent a measurable number of BDs cases. Therefore, our findings provide a quantitative scientific basis for formulating targeted environmental policies and optimizing the allocation of perinatal healthcare resources.

The identification of PM_2.5_ as the key pollutant in the overall population by QGC was corroborated by single-pollutant logistic regression models, in which PM2.5 also exhibited the largest effect estimate. Substantial evidence indicates that PM_2.5_ can disrupt normal embryonic development through complex, multi-system mechanisms. For instance, exposure interferes with embryonic hematopoiesis by downregulating key transcription factors such as SOX2, which leads to reduced hematopoietic stem cell numbers and function (33). Concurrently, PM_2.5_ poses a substantial threat to cardiac development. Specifically, extractable organic components can activate the aryl hydrocarbon receptor, trigger abnormal m6A RNA methylation, and induce mitochondrial dysfunction, thereby inhibiting cardiomyocyte differentiation and heart structure formation (34, 35). Moreover, PM_2.5_ disturbs placental metabolic homeostasis and impairs its nutrient-transport capacity, which may indirectly compromise fetal development (36). Therefore, future pollution control strategies should maintain a sustained focus on the coordinated management of PM_2.5_ while addressing other region-specific pollutants.

BKMR analysis further suggested complex interaction patterns between individual pollutants and the risk of BDs under mixed exposure conditions. PM_2.5_ and O_3_ exhibited stable and significant positive associations across background exposure levels. This stability indicates that their toxic effects likely operate through relatively independent pathophysiological pathways, are less susceptible to fluctuations in coexisting pollutant concentrations, and therefore constitute a consistent risk within common exposure ranges. In contrast, PM_10_ and SO_2_ showed significant positive associations only when other pollutants were present at high levels. This pattern raises the possibility that their individual effects may be weak or latent but can become apparent or enhanced in a high-concentration multi–pollutant environment, possibly through synergistic mechanisms such as the joint exacerbation of oxidative stress and inflammatory responses. NO_2_ also tended to show stronger associations at higher background exposure levels. Collectively, these findings underscore that the health effects of pollutants are not simply additive; rather, the risk may be synergistically amplified as the overall pollution burden increases (37–39). The analysis of two–pollutant E-R functions suggested potential interactions between PM_10_-SO_2_, NO_2_-SO_2_, and NO_2_-O_3_, supporting the synergy hypothesis. Consequently, in areas with severe air pollution, controlling single pollutants alone may be insufficient to effectively reduce BDs risk, highlighting the public health importance of implementing integrated multi–pollutant control strategies. It is noteworthy that CO displayed a significant negative association in the BKMR model. This apparent protective effect warrants cautious interpretation: it may reflect complex atmospheric dynamics or correlations with emission sources, or it may indicate residual confounding from unmeasured socioeconomic or behavioral factors, or suggest biological antagonism within the multi-pollutant mixture (31, 40, 41). Future research is warranted to clarify the specific chemical and biological mechanisms underlying these interactions.

Existing evidence suggests that residential area and offspring sex may act as effect modifiers in the association between air pollution and BDs (6, 42). In our subgroup analysis, although interaction terms were not statistically significant, heterogeneous risk patterns emerged. A significant positive joint effect of the pollutant mixture was observed exclusively in rural and female offspring subgroups, with O_3_ and SO_2_ identified as the key drivers, respectively. In contrast, PM_2.5_ remained the primary driver in urban and male offspring subgroups, consistent with the overall population, reflecting its role as a pervasive regional background pollutant. These variations likely arise from the interplay of environmental exposure profiles, biological susceptibilities, and socio-behavioral factors. First, rural and urban areas differ markedly in O_3_ precursor sources, such as crop residue burning and agricultural machinery emissions in rural settings versus traffic and industrial emissions in urban areas, which may impart distinct toxicity profiles to secondary pollutants (43, 44). Additionally, limited healthcare access and health risk management capacity in rural communities may heighten maternal susceptibility to specific pollutants (1). Second, sex-specific developmental mechanisms may explain the SO_2_-driven risk in female offspring. SO_2_ and its derivatives can induce oxidative stress, epigenetic modifications, and disrupted cellular signaling (45, 46). Animal studies further suggest sexual dimorphism in placental function, metabolic enzyme activity, and epigenetic regulation, potentially rendering female fetuses more sensitive to certain pollutants (47).

The key strength of this study is the integrated application of several advanced modeling frameworks, including BKMR, QGC, WQS, and ANR-based logistic regression, within a representative heavily industrialized city in China. This multi-model approach systematically evaluates the association of multi-pollutant mixture exposure on BDs risk. By more effectively handling the high collinearity among pollutants and quantifying the contributions of individual components, these methods overcome major limitations of traditional single-pollutant models. Thus, the study provides new epidemiological evidence to clarify the complex relationship between exposure to mixed air pollutants during the preconception and early pregnancy period and BDs risk.

Several limitations should be considered when interpreting the findings of this study. First, exposure assessment was based on IDW spatial interpolation using fixed-site monitoring stations. This method did not account for meteorological factors such as wind direction, wind speed, or atmospheric stability, which influence pollutant dispersion and transport, and may have introduced exposure misclassification bias. Additionally, residential address was used as the proxy for personal exposure, which did not capture variations related to daily mobility patterns or occupational locations, thereby introducing measurement error. Such non-differential misclassification tends to bias effect estimates toward the null and thus may underestimate the true associations (48, 49). It should be noted that this study included only six routinely monitored air pollutants, excluding other potentially toxic industrial pollutants (e.g., heavy metals, volatile organic compounds, polycyclic aromatic hydrocarbons, etc.), as these are not covered by fixed-site monitoring networks, precluding individual-level exposure estimates. Second, as a retrospective cohort study, the potential for selection bias due to missing information cannot be fully ruled out, although sensitivity analyses partially supported the robustness of the results. Furthermore, although we adjusted for numerous demographic and perinatal confounders, residual confounding may persist from unmeasured factors such as maternal folic acid supplementation, parental smoking, genetic susceptibility, and exposure to other environmental teratogens. Third, the multiple comparisons conducted across pollutants and outcomes increase the likelihood of spurious findings. Although we leveraged the complementarity of multiple statistical approaches and conducted sensitivity analyses, some significant associations may still reflect type I error. We therefore interpret our findings cautiously. Finally, BDs surveillance was limited to the period from 28 weeks of gestation to 7 days after birth. Cases diagnosed later in life were not captured, which may have led to an underestimation of the total disease burden attributable to pollutant exposure. Future studies with prospective, multi-center designs incorporating individual-level exposure assessment at higher spatiotemporal resolution (e.g., using land-use regression models or personal monitors), along with more comprehensive data on genetic, behavioral, and environmental co-exposures, would help to better elucidate the association between air pollutants and BDs.

## 5 Conclusions

In conclusion, this study suggests that periconceptional exposure to mixed air pollutants is associated with an increased risk of BDs, with PM_2.5_, SO_2_, and O_3_ identified as the principal drivers of this joint effect. These findings underscore the potential importance of reducing air pollution exposure during this critical developmental window to prevent BDs. The results support the development of protective strategies for pregnant women, strengthening comprehensive BDs prevention and informing the allocation of related healthcare resources. Future research is needed to investigate the effects of multi-pollutant exposure on BDs across specific organ systems and to explore the underlying biological mechanisms.

## Data Availability

The raw data supporting the conclusions of this article will be made available by the authors, without undue reservation.
