# Supplementary material for: The association between prenatal exposure to mixed air pollutants and birth defects risk: a population-based study in Tangshan, China
Source: Front Public Health. 2026 Apr 13;14:1791132. doi: 10.3389/fpubh.2026.1791132 (PMC13111202; doi:10.3389/fpubh.2026.1791132)
Supplement: Supplementary file 1 [file Data_Sheet_1.docx]

***Supplementary Material***

**Supplementary Figures and Tables**

Supplementary FIGURE S1 Spatial distribution of ambient air quality monitoring stations in Tangshan, China

Supplementary FIGURE S2 Location of participants in Tangshan, China

Supplementary FIGURE S3 Schematic diagram of the exposure window

Supplementary FIGURE S4 Spearman’s correlation coefficients among air pollutants during the exposure assessment period

Supplementary FIGURE S5 Exposure-response curve of single-pollutant exposure and BDs risk using restricted cubic spline (RCS)

Supplementary FIGURE S6 Joint effect for BDs risk using quantile g-computation (QGC) by subgroup

Supplementary FIGURE S7 Association of mixed air pollutants exposure with isolated BDs risk using Bayesian kernel machine regression (BKMR)

Supplementary FIGURE S8 Daily level of air pollutants, Tangshan, China (September 2019-June 2022)

Supplementary TABLE S1 Descriptive statistics of air pollutants, Tangshan, China (September 2019-June 2022)

Supplementary TABLE S2 Birth defect (BD) codes by category

Supplementary TABLE S3 Association of single-pollutant exposure with BDs risk using logistic regression

Supplementary TABLE S4 Association of exposure to mixed air pollutants with the risk of BDs


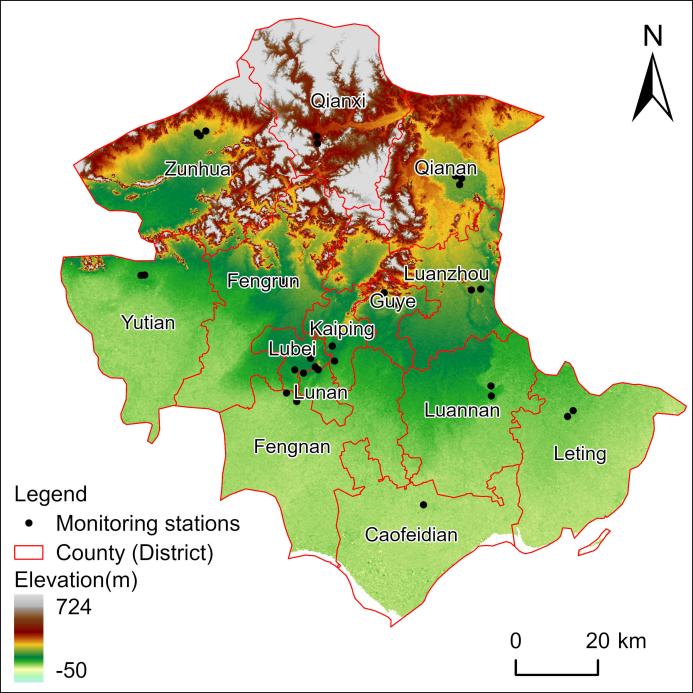


Supplementary FIGURE S1

Spatial distribution of ambient air quality monitoring stations in Tangshan, China.


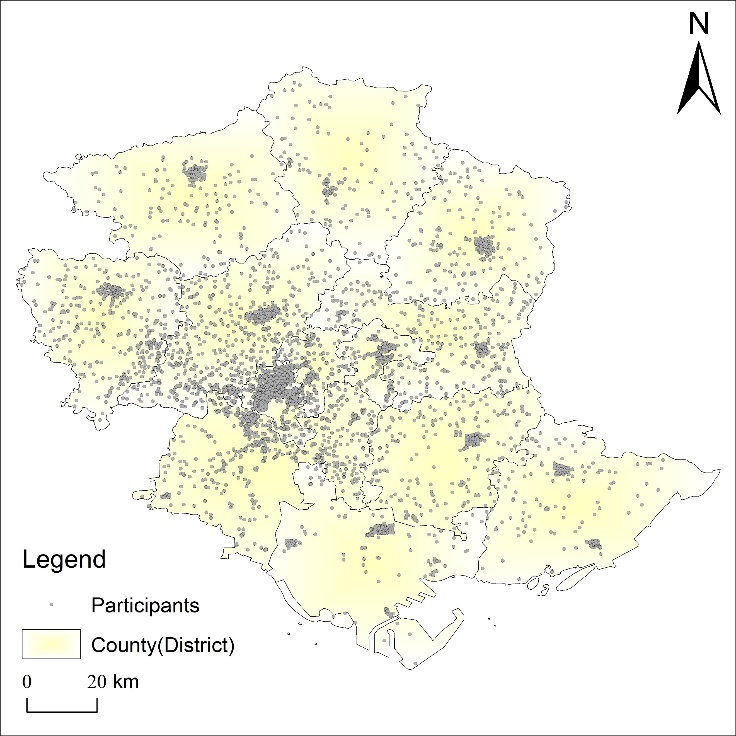


Supplementary FIGURE S2

Location of participants in Tangshan, China.


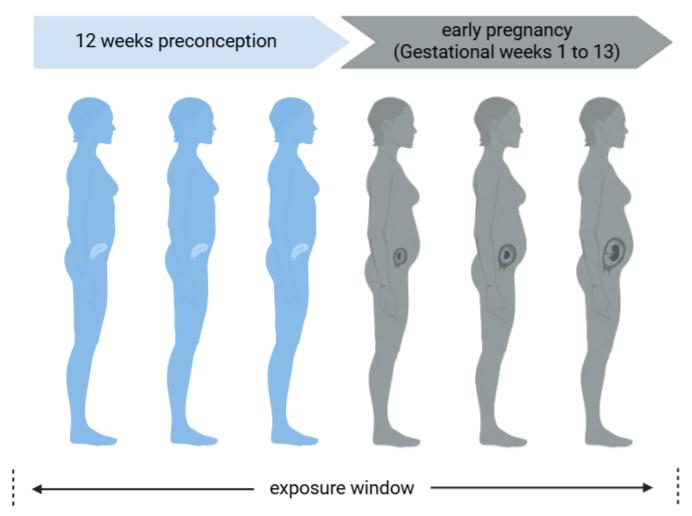


Supplementary FIGURE S3

Schematic diagram of the exposure window.


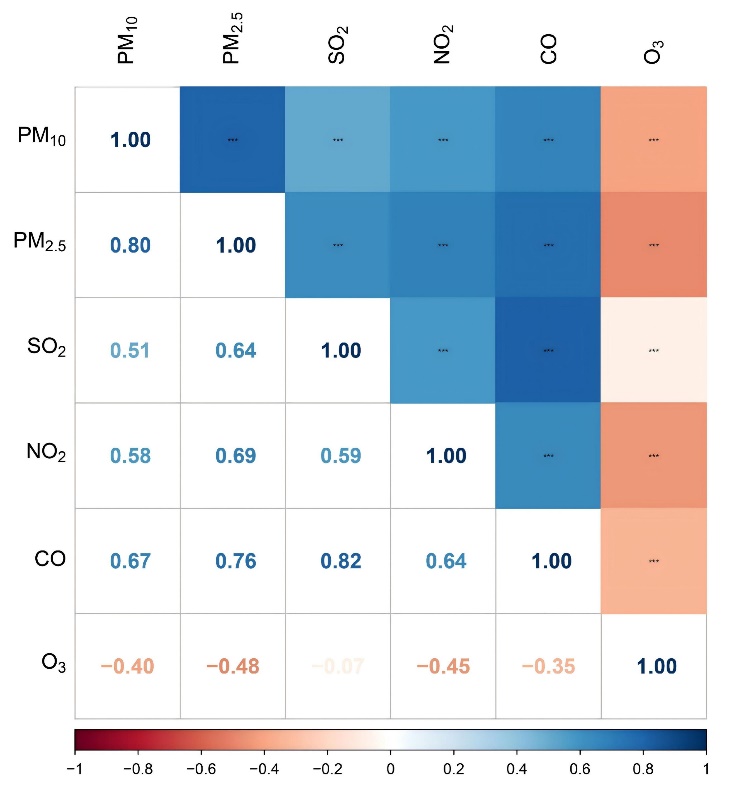


Supplementary FIGURE S4
Spearman’s correlation coefficients among air pollutants during the exposure assessment period.

***P < 0.001.


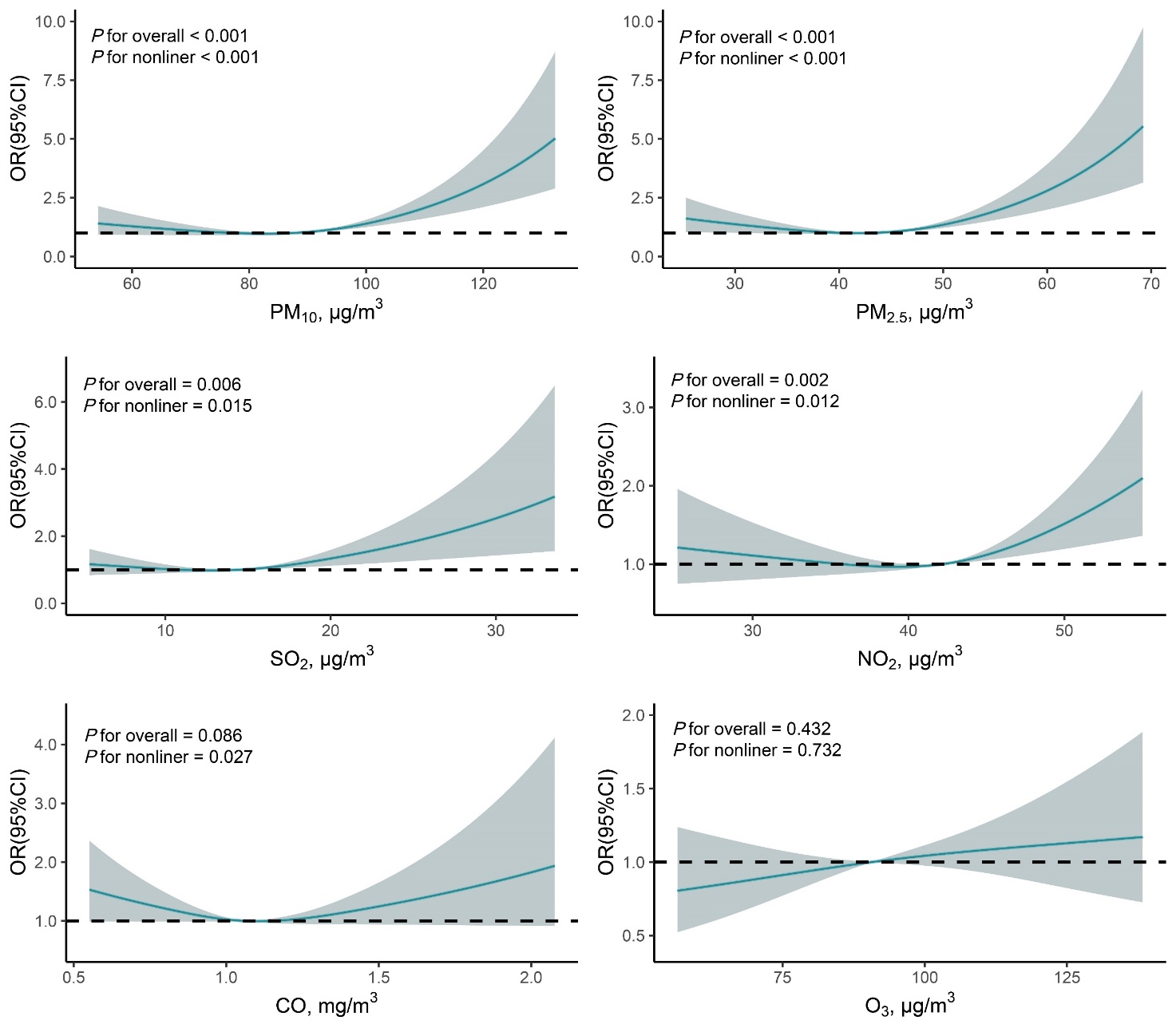


Supplementary FIGURE S5
Exposure-response curve of single-pollutant exposure and BDs risk using restricted cubic spline (RCS).

Models were adjusted for maternal age, educational level, employment status, residential area, parity, season of conception, gestational age, offspring sex. BDs, birth defects.


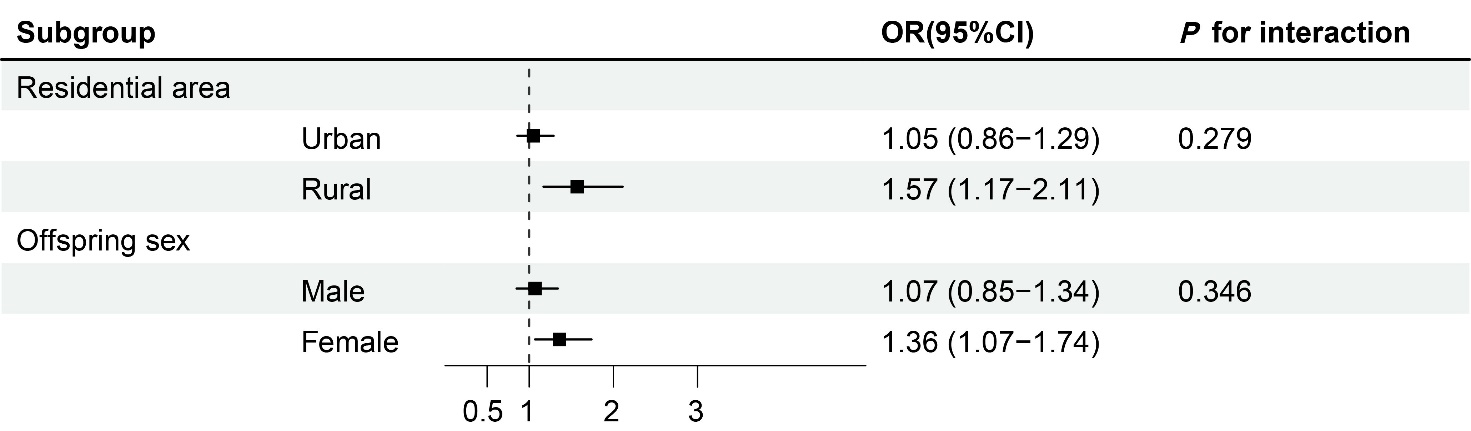


Supplementary FIGURE S6

Joint effect for BDs risk using quantile g-computation (QGC) by subgroup.

Models stratified by residential area were adjusted for maternal age, educational level, employment status, parity, season of conception, gestational age, offspring sex. Models stratified by offspring sex were adjusted for maternal age, educational level, employment status, residential area, parity, season of conception, gestational age. OR (95%CI) per quartile increase in multi-pollutant mixture was estimated. BDs, birth defects; OR, odds ratio; CI, confidence interval.


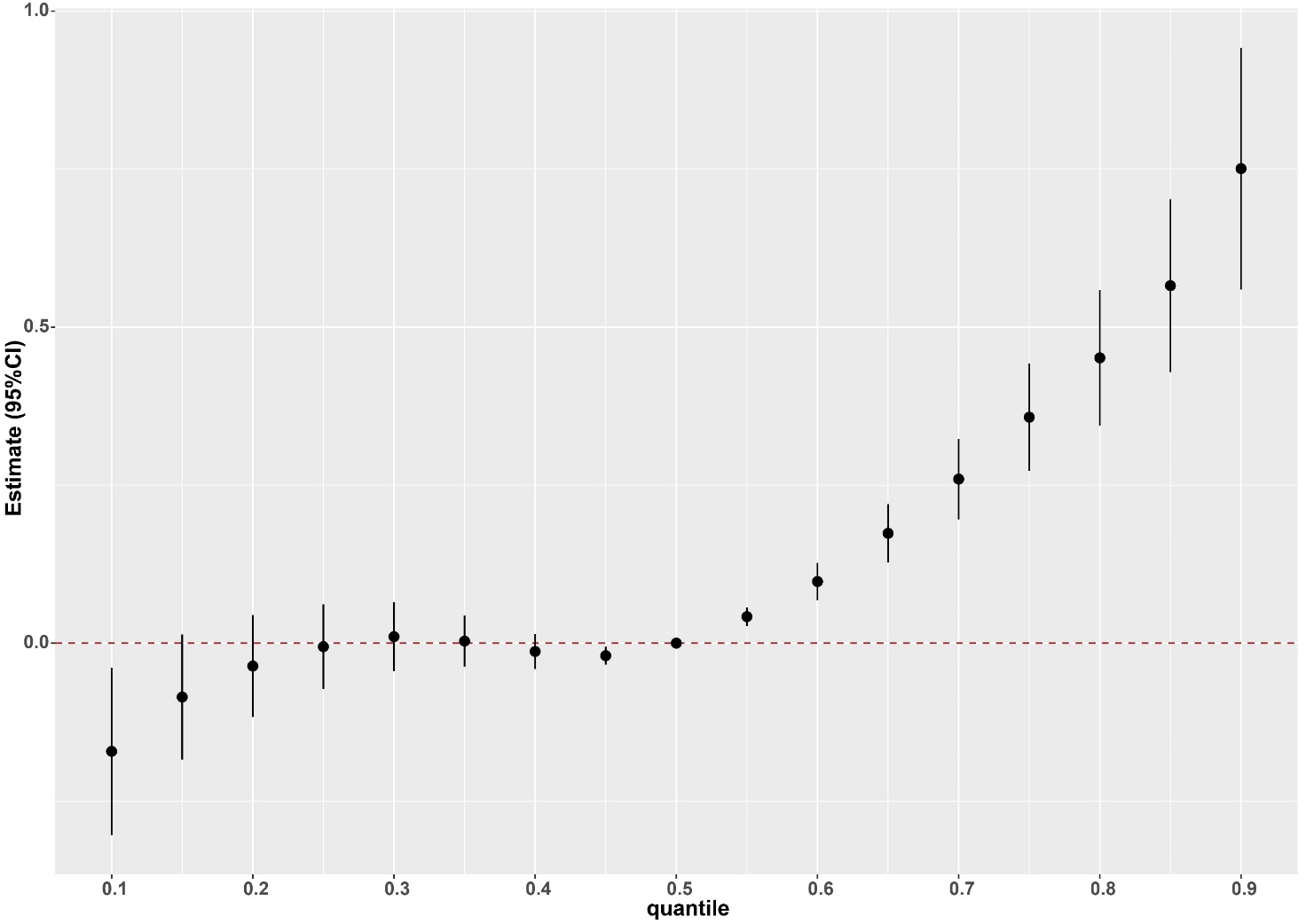


Supplementary FIGURE S7

Association of mixed air pollutants exposure with isolated BDs risk using Bayesian kernel machine regression (BKMR).

Air pollutants concentrations were ln-transformed and normalized. The estimated change of multi-pollutant mixture exposure on BDs risk when all air pollutants were at a particular quantile compared to *P*_50_. Models were adjusted for maternal age, educational level, employment status, residential area, parity, season of conception, gestational age, offspring sex. BDs, birth defects; CI, confidence interval.


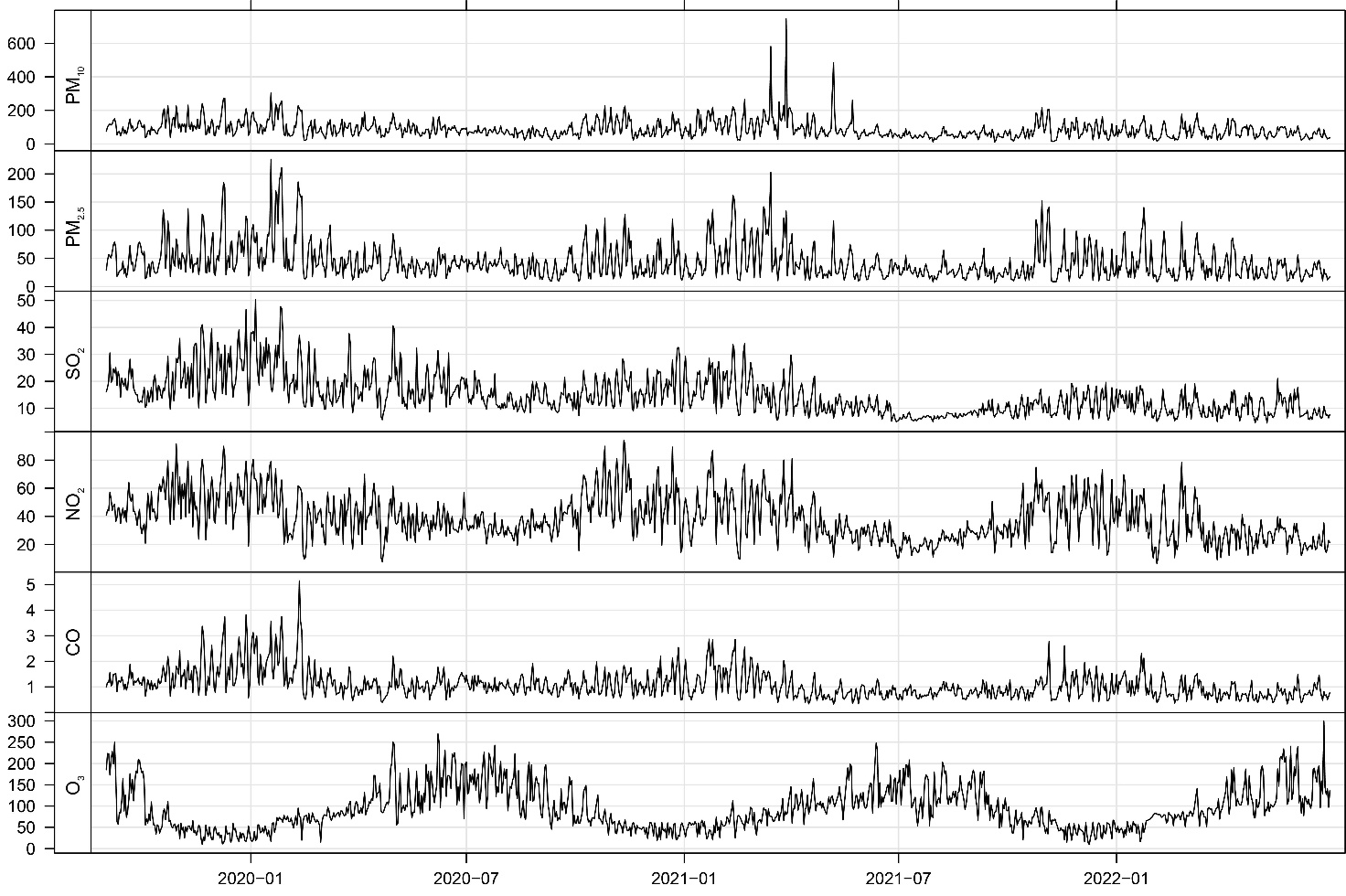


Supplementary FIGURE S8

Daily level of air pollutants, Tangshan, China (September 2019-June 2022).

Units were µg/m^3^ for PM_10_, PM_2.5_, SO_2_, NO_2_, and O_3_, mg/m^3^ for CO.

Supplementary TABLE S1 Descriptive statistics of air pollutants, Tangshan, China (September 2019-June 2022)

| Air pollutant | Mean | SD | *Min* | *P_25_* | *P_50_* | *P_75_* | *Max* |
| --- | --- | --- | --- | --- | --- | --- | --- |
| PM_10_, µg/m^3^ | 89.07 | 55.75 | 11.00 | 52.72 | 76.98 | 110.03 | 745.78 |
| PM_2.5_, µg/m^3^ | 44.02 | 31.99 | 6.90 | 22.37 | 35.30 | 54.58 | 225.83 |
| SO_2_, µg/m^3^ | 14.86 | 7.43 | 4.53 | 9.27 | 13.18 | 18.32 | 50.24 |
| NO_2_, µg/m^3^ | 38.92 | 16.29 | 6.57 | 27.01 | 36.10 | 48.96 | 94.03 |
| CO, mg/m^3^ | 1.10 | 0.56 | 0.33 | 0.71 | 0.98 | 1.30 | 5.15 |
| O_3_, µg/m^3^ | 91.34 | 51.22 | 9.76 | 56.40 | 83.35 | 121.83 | 299.33 |

Notes: SD, standard deviation.

Supplementary TABLE S2 Birth defect (BD) codes by category

| Categories | | Code Range |
| --- | --- | --- |
| Nervous system malformations | Brain | Q00-Q04 |
|  | Spine | Q05-Q06 |
|  | Other | Q07 |
| Head and neck malformations | Eye, ear, face and neck | Q10-Q18 |
| Circulatory system malformations | Congenital heart disease | Q20-Q24 |
|  | Arteries | Q25-026 |
|  | Peripheral circulatory system | Q27-Q28 |
| Congenital respiratory system malformations | | Q30-Q34 |
| Cleft lip, cleft palate | | Q35-Q37 |
| Digestive system malformations | | Q38-Q45 |
| Reproductive organs malformations | Female | Q50-Q52 |
|  | Male | Q53-Q55 |
|  | Unclear gender and pseudohermaphroditism | Q56 |
| Urinary system malformations | | Q60-Q64 |
| Musculoskeletal system malformations | | Q65-Q79 |
| Other congenital malformations | | Q80-Q89 |
| Chromosomal abnormalities | | Q90-Q99 |

Supplementary TABLE S3 Association of single-pollutant exposure with BDs risk using logistic regression

| Air pollutant | Model 1 | |  | Model 2 | |
| --- | --- | --- | --- | --- | --- |
|  | OR (95%CI) | *P*-value |  | OR (95%CI) | *P*-value |
| PM_10_ | 1.00 (0.94–1.06) | 0.965 |  | 1.14 (1.06–1.23) | < 0.001 |
| PM_2.5_ | 1.01 (0.90–1.13) | 0.887 |  | 1.25 (1.10–1.45) | < 0.001 |
| SO_2_ | 1.09 (0.90–1.32) | 0.385 |  | 1.22 (0.99–1.50) | 0.056 |
| NO_2_ | 1.10 (0.94–1.27) | 0.318 |  | 1.22 (1.04–1.42) | 0.015 |
| CO | 0.95 (0.91–0.99) | 0.013 |  | 0.99 (0.95–1.05) | 0.919 |
| O_3_ | 1.06 (1.02–1.10) | 0.005 |  | 1.05 (0.97–1.12) | 0.211 |

Notes: Model 1 was crude model; Model 2 was adjusted for maternal age, educational level, employment status, residential area, parity, season of conception, gestational age, offspring sex. OR (95%CI) was estimated for a 10 µg/m^3^ increment in PM_10_, PM_2.5_, SO_2_, NO_2_, and O_3_, and a 0.1mg/m^3^ increment in CO. BDs, birth defects; OR, odds ratio; CI, confidence interval.

Supplementary TABLE S4 Association of exposure to mixed air pollutants with the risk of BDs

| Multi-pollutant statistical model | OR | 95% CI | *P-*value |
| --- | --- | --- | --- |
| Quantile g-computation (PM_10_, PM_2.5_, SO_2_, NO_2_, CO, and O_3_) ^a^ | 1.19 | 1.01–1.41 | 0.039 |
| Quantile g-computation (PM_10_, PM_2.5_, SO_2_, NO_2_, CO, and O_3_) ^b^ | 1.17 | 0.99–1.38 | 0.064 |
| Weighted quantile sum regression (PM_10_, PM_2.5_, SO_2_, NO_2_, CO, and O_3_) | 1.21 | 1.04–1.39 | 0.011 |
| Adaptive network regularization combined with logistic regression | 1.35 | 1.22–1.50 | <0.001 |

Notes: Models were adjusted for maternal age, educational level, employment status, residential area, parity, season of conception, gestational age, offspring sex. OR (95%CI) was estimated per quartile increase in the mixture [quantile g-computation (QGC) and weighted quantile sum (WQS) regression] and per SD increase in RS [adaptive network regularization (ANR) with logistic regression]. BDs, birth defects; OR, odds ratio; CI, confidence interval; SD, standard deviation; RS, risk score. ^a^ QGC for total BDs; ^b^ QGC for isolated BDs.
